# Interposition versus transposition technique in microvascular decompression for trigeminal neuralgia secondary to vertebrobasilar dolichoectasia: a systematic review and pooled meta-analysis

**DOI:** 10.3389/fneur.2024.1474553

**Published:** 2024-11-25

**Authors:** Francesco Signorelli, Fabio Zeoli, Valid Rastegar, Flavia Beccia, Riccardo Caronna, Massimiliano Visocchi

**Affiliations:** ^1^Department of Neurosurgery, Fondazione Policlinico Universitario “A. Gemelli” IRCCS, Rome, Italy; ^2^Università Cattolica del Sacro Cuore, Rome, Italy; ^3^Department of Life Sciences and Public Health, Fondazione Policlinico Universitario “A. Gemelli” IRCCS, Rome, Italy

**Keywords:** microvascular decompression, vertebrobasilar dolichoectasia, trigeminal neuralgia, interposition, transposition

## Abstract

**Introduction:**

Limited data are available comparing the interposition and transposition techniques for microvascular decompression (MVD) in patients with trigeminal neuralgia (TN) secondary to vertebrobasilar dolichoectasia (VBD); this study aims to review current findings on TN associated with VBD and compare the interposition and transposition techniques in terms of surgical morbidity and patient outcomes.

**Methods:**

Following the PRISMA guidelines, PubMed/Medline, Web of Science, and SCOPUS databases were searched to identify studies reporting patients undergoing MVD for TN secondary to VBD. The studies were divided into two groups, interposition and transposition, based on the microvascular decompression technique used. Studies not reporting the diagnostic criteria, included less than five cases, or were not available in English were excluded.

**Results:**

Fourteen eligible papers were retrieved, of which five studies reported cases undergoing the interposition technique, eight studies for the transposition technique, and one study reported cases from both groups. Data including preoperative and postoperative BNI class, comorbidities, and postoperative complications were retrieved to analyze and compare the two techniques in terms of efficacy and long-term outcomes in treating TN secondary to VBD.

**Conclusion:**

Both interposition and transposition techniques for MVD yield high rates of pain relief in patients with TN secondary to VBD. While both approaches demonstrate similar efficacy, the interposition method is associated with a lower rate of long-term complications. Further research, preferably through randomized prospective studies, is needed to refine surgical strategies and improve patient outcomes.

## Introduction

Trigeminal neuralgia (TN) is a common chronic pain disorder characterized by recurrent episodes of electric shock-like or stabbing pain affecting the dermatomal distribution of trigeminal nerve branches (1, 2). Between 80 and 90% of the cases of TN are caused by a neurovascular conflict where the trigeminal nerve is compressed by an adjacent artery or a vein, with the superior cerebellar artery being the most implicated vessel (1, 3). Less commonly TN is secondary to vertebrobasilar dolichoectasia (VBD), a rare cerebrovascular abnormality characterized by an ectatic, elongated, and tortuous vertebrobasilar artery (VBA) complex (4). These abnormal vessels may sometimes compress, directly or indirectly—thorough displacements of adjacent vessels (5)—the root of the trigeminal nerve resulting in TN (6). Recent published studies report VBD-induced TN accounting for 2–7.7% of all cases of TN (1, 7–10).

At present, there is a global consensus that surgical intervention through microvascular decompression (MVD) is recommended for drug-resistant TN when an offending vessel causing neurovascular compression can be identified. In VBD-induced TN cases, given the unusual anatomy due to the wide and abnormally located vertebrobasilar artery complex, surgical risks are higher. Two techniques can be considered to address this condition: the interposition method and the transposition method. The former represents the standard MVD approach, routinely used in cases of classical TN due to neurovascular conflict; it requires the insertion of implants between the nerve and the offending vessel. The latter entails repositioning the VBA complex using various materials such as aneurysm clips, biomedical glues, Prolene sutures, tapes, and titanium plates. To date, current literature on this topic is scarce, with only one available study that directly compares the two techniques. The purpose of this systematic review and meta-analysis is to review current findings on TN associated with VBD and compare the two techniques in terms of surgical morbidity and postoperative outcomes.

## Materials and methods

### Search strategy

The systematic review was performed according to Preferred Reporting Items for Systematic Reviews and Meta-Analyses (PRISMA) 2020 guidelines (11) to investigate the outcomes of Microvascular decompression in patients with trigeminal neuralgia secondary to vertebrobasilar dolichoectasia (VBD) and compare the interposition technique to the transposition technique. A comprehensive literature search of PubMed, Web of Science, and Scopus was performed on for studies published in English from January 1991 to February 2024. Our systematic review was registered and accepted in PROSPERO database with the following ID: CRD42024523971. The keywords and the detailed search strategy are reported in Supplementary File 1. After searching the three databases, all results were collected. Duplicates were removed using Rayyan software (12). All remaining articles were then fully screened by 3 reviewers (FZ, VR, RC); a senior author (FS) resolved discrepancies.

### Data extraction

From each study, the following data were extracted: author/s; year of publication; study design, number of patients enrolled; demographic data; mean follow-up time; patient comorbidities; reported prevalence of VBD-induced trigeminal neuralgia; trigeminal neuralgia characteristics (side and branches involved); additional vessels compressing the trigeminal nerve in addition to the VBA; postoperative complications (transient and permanent); preoperative BNI; postoperative BNI and BNI at last follow up.

### Statistical analysis

Meta-regression and meta-analysis of proportions with binomial distribution were used to assess the effect of MVD transposition and interposition post-operatively and at the last follow-up. The pooled prevalence of BNI was calculated using the inverse variance method, adopting fixed effects models if the tests met the hypothesis of homogeneity, or random effects model otherwise (13). Heterogeneity across the included studies was analyzed using the Q test and the I^2^ index (values of 25, 50, and 75% are taken as low, medium, and high heterogeneity, respectively) (14). Subgroup analyses were performed by the intervention type. A forest plot was used to present the pooled prevalence. The leave-one-out sensitivity test was used to confirm that the findings were not driven by any single study. In addition, Egger’s tests were used to detect potential publication bias by examining the funnel plot symmetry. The odds ratio (OR) for the development of postoperative complications in both cohorts, including 95% confidence intervals (CI), was calculated. A *p*-value <0.05 indicated statistical significance. Statistical analyses were performed using STATA18 software.

### Quality scoring

Three authors (FZ, VR, and RC) independently assessed the risk of bias for each included study using the Risk of Bias In Non-randomized Studies—of Interventions (ROBINS-I) tool (15). The quality of evidence for outcomes was assessed by the Joanna Briggs Institute (JBI) critical appraisal checklist (16).

### Outcomes

The primary outcomes of this systematic review and meta-analysis were the following: (1) to determine the prevalence and clinical features of TN secondary to VBA, and (2) to analyze the different surgical strategies and related clinical outcomes and complication rate. We compared the efficacy of interposition and transposition methods by assessing the Barrow’s Neurological Institute (BNI) grade (17) (Table 1) post-operative and at last follow-up. BNI grades I–II were considered adequate pain relief, whereas BNI grades III–V indicated pain recurrence.

**Table 1:** Barrow Neurological Institute (BNI) pain intensity score (17).

Score	
I	No trigeminal pain, no medication
II	Occasional pain, not requiring medication
III	Some pain, adequately controlled with medication
IV	Some pain, not adequately controlled with medication
V	Severe pain or no pain relief

## Results

### Literature review

**Figure 1:**
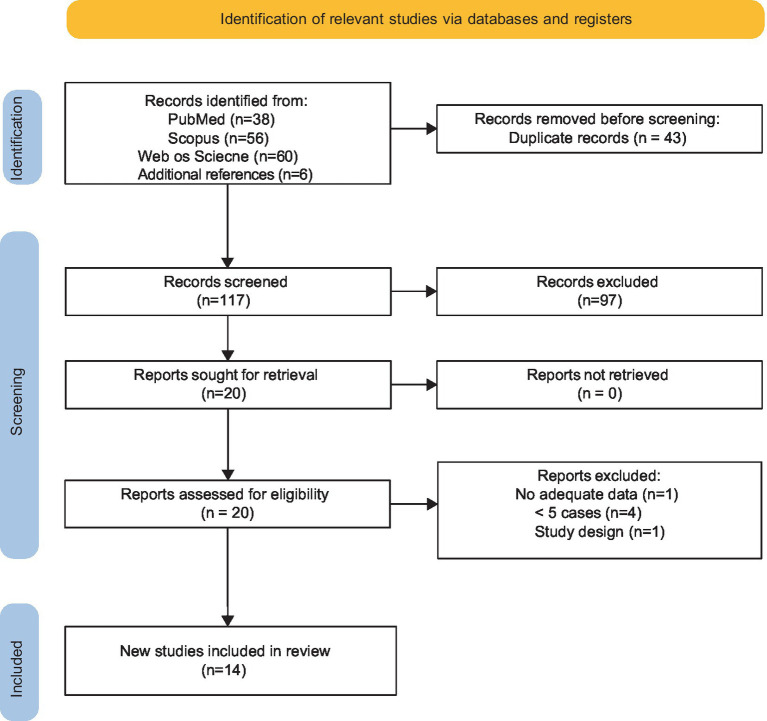
PRISMA diagram showing the details of the systematic search of the literature.

The search strategy yielded 160 results. After the removal of duplicates, articles were screened by title and abstract for relevance. The remaining articles were then screened via full text (see the PRISMA diagram shown in Figure 1). Studies meeting the defined criteria were included for quantitative analysis. The characteristics of the individual studies are presented in Table 2. Fourteen studies including 306 patients with trigeminal neuralgia secondary to VBD were analyzed in this review. All included studies presented a retrospective single-center design. The exclusion criteria were the following: (1) studies with less than five patients; (2) case reports; (3) review articles; (4) technical notes; (5) studies published in languages other than English with no available English translations; (6) case series not dealing with trigeminal neuralgia caused by VBA conflict; (7) case series with no specific data on surgical steps performed. Furthermore, we only included studies in which VBD was defined according to specific diagnostic criteria as first proposed by Ubogu and Zaidat (18): basilar artery (BA) or vertebral artery (VA) diameter > 4.5 mm, deviation of any portion >10 mm from the shortest expected course, BA length > 29.5 mm or intracranial VA length > 23.5 mm, BA bifurcation above the suprasellar cistern or any BA portion lying adjacent to the margin of the clivus or dorsum sellae (19).

**Table 2:** Summary of the studies included in the meta-analyses.

References	Study design	Type of intervention	M/F	Follow-up (mean, months)
Zheng et al. (5)	R	VBA transposition	31/30	24.5
Amagasaki et al. (25)	R	VBA transposition	12/20	37.9
Inoue et al. (20)	R	VBA transposition	13/13	47
Liu et al. (21)	R	VBA transposition	9/13	22
Wang et al (22)	R	VBA transposition	9/14	32
Vanaclocha et al. (23)	R	VBA transposition	5/3	56.5
Linskey et al. (24)	R	VBA transposition	21/10	60
Yang et al. (10)	R	VBA transposition	5/5	
Honey and Kaufmann (29)	R	Interposition	1/1	68.3
		VBA transposition	9/2	
Yu et al. (6)	R	Interposition	21/9	76.67
Shulev et al. (26)	R	Interposition	4/10	66
Sun et al. (27)	R	Interposition	8/7	29.8
Ma et al. (9)	R	Interposition	8/3	22
El-Ghandour (28)	R	Interposition	6/4	93.6

### Demographic data and risk factors

In our meta-analysis, the reported prevalence of vertebrobasilar dolichoectasia (VBD) among patients who underwent microvascular decompression (MVD) for trigeminal neuralgia ranged from 2 to 7% with a mean prevalence of 4%.

The mean age of patients varied between 54 and 68 years, with males comprising 53% (95% CI: 47–59%) of the cohort. Hypertension was reported in 59% (95% CI: 52–68%) of the patients. The right side was affected in 43% (95% CI: 37–48%) of patients, with the V2 branch being the most frequently involved, accounting for 82% (95% CI: 78–87%) of cases. The most common clinical presentation was the involvement of both V2 and V3 branches (43, 95% CI: 37–48%). Detailed data are reported in Table 3.

**Table 3:** Demographic data and clinical characteristics.

	N of patients	%	95% CI
Males	162/306	52.94%	47–59%
Reported prevalence of TN secondary to VBD	304/7829	3.88%	3–4%
Comorbidities			
Hypertension	121/205	59.02%	52–66%
Type 2 diabetes	10/205	4.88%	2–8%
Stroke	7/205	3.41%	1–6%
Trigeminal neuralgia characteristics			
Right side involvement	131/306	42.81%	37–48%
V1 involvement	63/306	20.59%	16–25%
V2 involvement	252/306	82.35%	78–87%
V3 involvement	202/306	66.01%	61–71%
V1V2	27/306	8.82%	6–12%
V1V3	1/306	0.33%	0–1%
V2V3	131/306	42.81%	37–48%
V1V2V3	26/306	8.50%	5–12%
VII involvement			
Hemifacial spasm	16/306	5.23%	3–8%
Vessel responsible for compression			
AICA	93	30.39%	25–36%
SCA	77	25.16%	20–30%
Vein	31	10.13%	7–14%
PICA	18	5.88%	3–9%
Trigeminocerebellar artery	2	0.65%	0–2%

### Surgical approach and intraoperative findings

In our analysis, we found the retrosigmoid suboccipital to be the most frequently used approach for MVD. Following this approach, the dura mater is incised along the sigmoid and transverse sinuses, then, after a gradual release of cerebrospinal fluid (CSF) from the lateral cerebellar cistern, the cerebellum is retracted medially to facilitate exposure. The arachnoid membrane is then dissected to provide full visualization of the lower cranial nerves.

Other than vertebrobasilar artery (VBA) compression, additional vessels were found to be compressing the trigeminal nerve. The anterior inferior cerebellar artery (AICA) and the superior cerebellar artery (SCA) were the most frequently reported additional offending vessels, involved in 30% (95% CI: 25–36%) and 25% (95% CI: 20–30%) of cases, respectively. Detailed data are reported in Table 3.

There were eight studies reporting patients treated with the transposition technique (5, 10, 20–25), and five studies reporting on the interposition technique (6, 9, 26–28). One case series included patients treated with either technique (29). In the transposition group (*n* = 224), VBA repositioning was achieved using various materials including Teflon sling/roll/pads/felt, biomedical glue, aneurysm clips, Ivalon sponge, autologous muscle, and silicon sheet. In the interposition group (*n* = 82) pieces of Teflon between the trigeminal nerve and the vessel responsible for the neurovascular conflict were placed, without forcing the repositioning of the vessel.

### Interposition vs. transposition: clinical outcomes and postoperative complications

**Figure 2:**
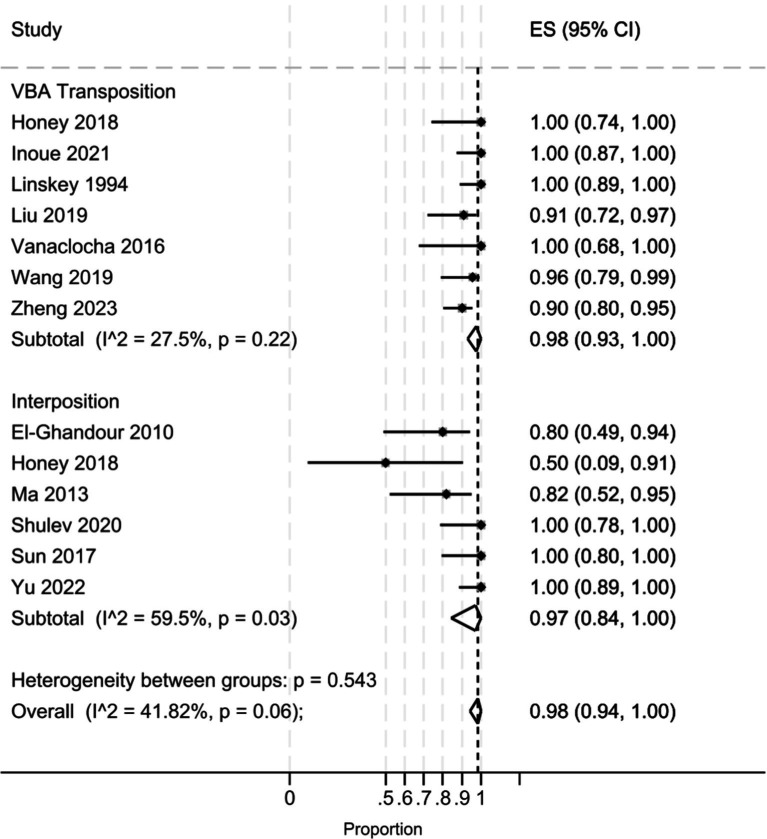
Forest plot detailing the pooled rate and 95% confidence intervals for the rate of post-operative BNI I-II in the VBA transposition group and the Interposition group.

**Figure 3:**
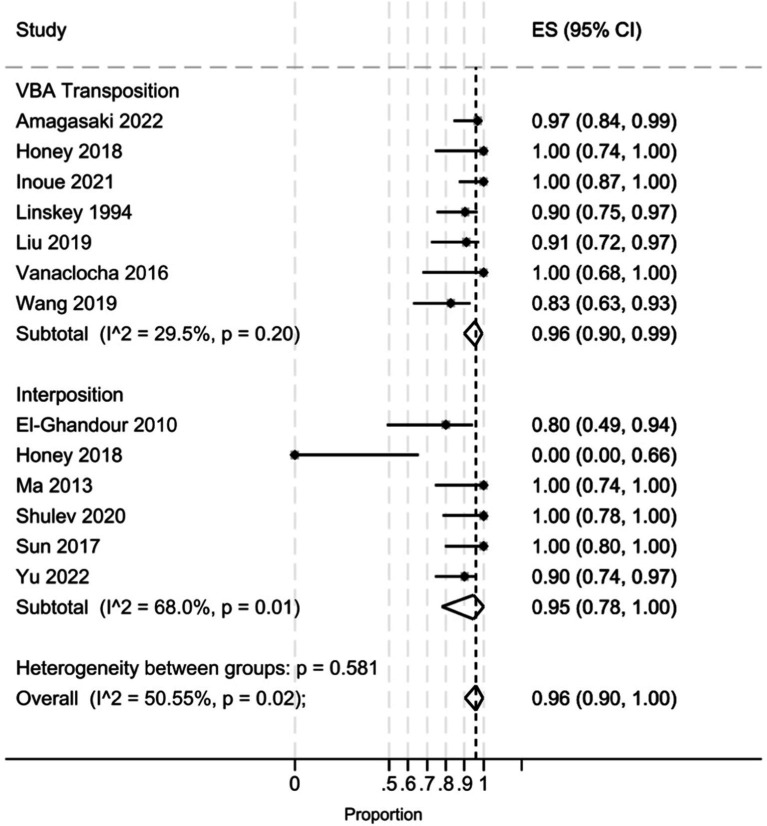
Forest plot detailing the pooled rate and 95% confidence intervals for the rate of BNI I-II in the VBA transposition group and the Interposition group at last follow-up.

**Figure 4:**
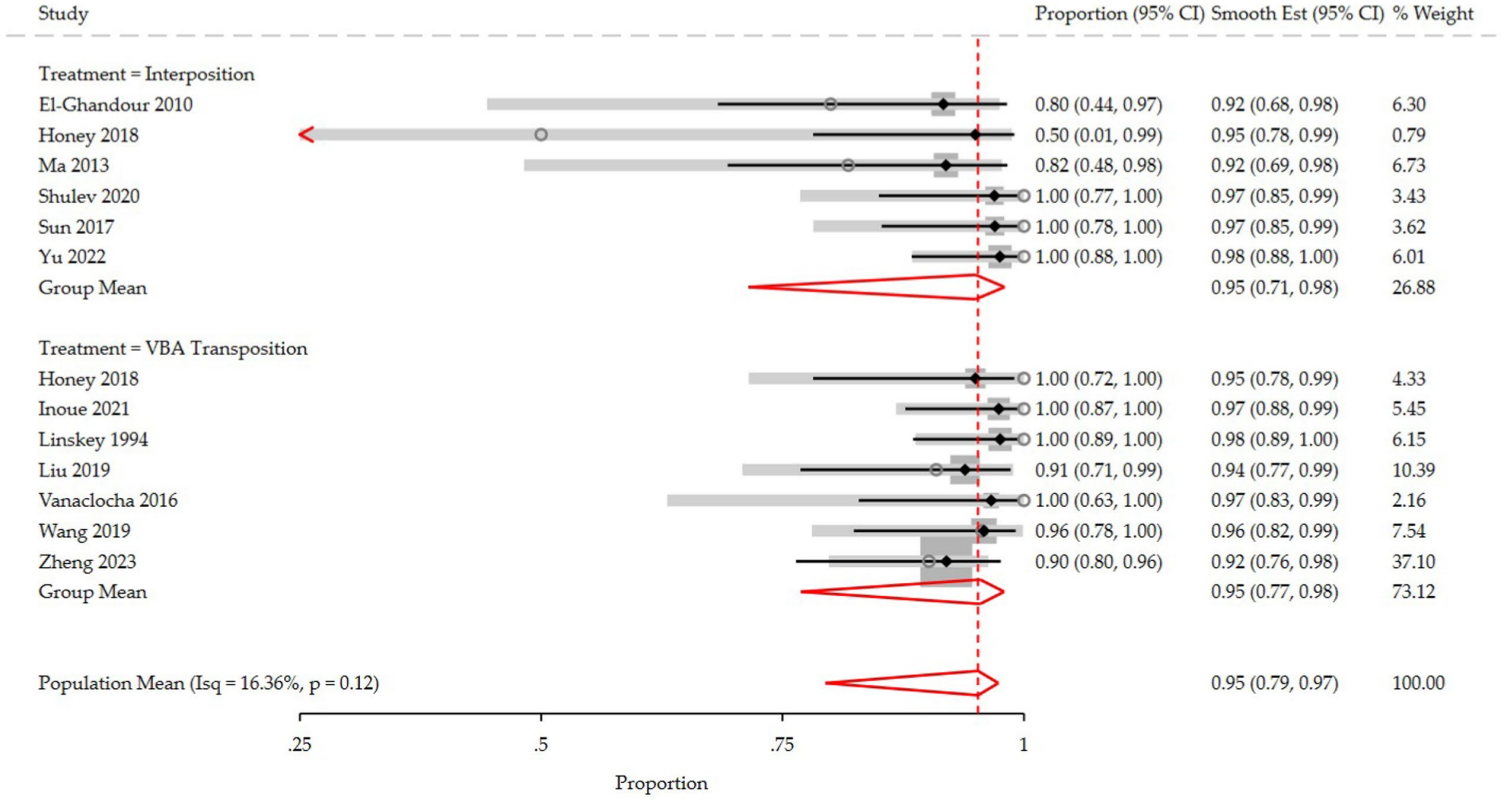
Forest plot detailing the meta-regression rate and 95% confidence intervals for the rate of post-operative BNI I-II in the VBA transposition group and the Interposition group.

The pooled analysis (Figure 2) demonstrated that the interposition approach resulted in post-operative Barrow Neurological Institute (BNI) grades I-II in 97% (95% CI: 84–100%, I^2^ = 59.5%, *p* = 0.03) of cases, compared to 98% (95% CI: 93–100%, I^2^ = 27.5%, *p* = 0.22) with the transposition approach. At the last follow-up, BNI I-II was observed in 95% (95% CI: 78–100%, I^2^ = 68%, *p* = 0.01) of patients treated with interposition and 96% (95% CI: 90–99%, I^2^ = 29.5%, *p* = 0.20) of those treated with transposition (Figure 3). After assessing the comparability of the two patient groups and stratifying by intervention type, the meta-regression estimated a BNI I-II rate of 96% for both the interposition group (95% CI: 71–98%) and the transposition group (95% CI: 77–98%) (Figure 4).

However, the interposition group showed a significantly lower pooled complication rate compared to the transposition group (4.88% vs 17.41%; OR 0.24, 95% CI 8-70%, *p* = 0.009). Early postoperative complications in the interposition group included facial weakness (10%, 95% CI: 3-16%), facial hypoesthesia (2%, 95% CI: 0-6%), and hearing impairment (2%, 95% CI: 0-6%). Notably, all cases of facial weakness resolved, while facial hypoesthesia and hearing loss persisted at the last follow-up.

In contrast, the transposition group had higher rates of complications: fifteen patients (7%, 95% CI: 3–10%) experienced permanent facial hypoesthesia, and seventeen patients (8%, 95% CI: 4–11%) reported permanent hearing impairment. Additionally, nine patients developed postoperative meningitis (4%, 95% CI: 1–7%). Details on the rate of postoperative complications are shown in Table 4.

**Table 4:** Postoperative complications.

	Interposition group				Transposition group			
	Raw data	(%)	CI 95%	Number of articles	Raw data	(%)	CI 95%	Number of articles
POSTOPERATIVE COMPLICATIONS								
CN IV deficit								
(Transient)	2	2,44%	0% - 6%	6	5	2,23%	0% - 4%	9
(Permanent)	0	0,00%		6	0			9
CN VI deficit								
(Transient)	1	1,22%	0% - 4%	6	15	6,70%	3% - 10%	9
(Permanent)	0	0,00%		6	1	0,45%	0% - 1%	9
Facial hypothesia								
(Transient)	0	0,00%		6	5	2,23%	0% - 4%	9
(Permanent)	2	2,44%	0% - 6%	6	15	6,70%	3% - 10%	9
Facial weakness								
(Transient)	8	9,76%	3% - 16%	6	8	3,57%	1% - 6%	9
(Permanent)	0	0,00%		6	5	2,23%	0% - 4%	9
Hearing loss/impairment								
(Transient)	0	0,00%		6	1	0,45%	0% - 1%	9
(Permanent)	2	2,44%	0% - 6%	6	17	7,59%	4% - 11%	9
Cerebellar Ataxia								
(Transient)	0	0,00%		6	4	1,79%	0% - 4%	9
(Permanent)	0	0,00%		6	1	0,45%	0% - 1%	9
Taste hypoestesia								
(Transient)	0	0,00%		6	0			9
(Permanent)	0	0,00%		6	0			9
Meningitis								
	0	0,00%		6	9	4,02%	1% - 7%	9
CSF leakeage								
	0	0,00%		6	1	0,45%	0% - 1%	9
Supratentorial acute subdural hematoma		2,44%						
	0	0,00%		6	1	0,45%	0% - 1%	9
TOTAL (Permanent)	4	4,88%	0% - 10%	6	39	17,41%	12% - 22%	9

## Discussion

Vertebrobasilar dolichoectasia (VBD; from the Greek dolicho, “elongated,” and ectasia, “dilated”) is an uncommon cause of trigeminal neuralgia (TN). It refers to a vascular abnormality characterized by expansion, elongation, and tortuosity of the vertebrobasilar system. Di Carlo et al. previously performed a meta-analysis and systematic review on VBD-related TN (30), and more recently, we published an updated narrative review on the topic (31). However, neither of these works included a quantitative comparison of the interposition and transposition techniques. The current study aims to address that gap by directly comparing these surgical approaches and evaluating their outcomes.

### Epidemiology and clinical characteristics

The etiopathogenesis of VBD is still largely unknown, but evidence points toward a multifactorial process combining congenital vascular wall abnormalities with acquired factors related to atherosclerosis (32, 33). Our analysis found that 52.94% of the population were males, and hypertension was present in 59.02% of cases. These findings align with the literature, indicating that patients with VBD-related TN are more likely to be older males with a history of hypertension, diabetes, hyperlipidemia, and myocardial infarction (6, 8, 23, 24, 28, 34). Addressing these risk factors postoperatively may improve long-term outcomes and potentially delay or prevent pain recurrence to a certain extent, though further research is needed to confirm this statement (5).

Our study found that the left side was more frequently affected (57.19%), consistent with previous findings (30). Left predominance may be related to hemodynamic and anatomical factors, as blood flow and shear stress are higher in the left VA than the right one (since the left subclavian artery, from which the VA arises, originates directly from the aortic arch), resulting in an asymmetric blood flow to the basilar artery with subsequent elongation and curvation of the VBA complex toward the weaker vertebral artery (6).

The V2 and V3 branches of the trigeminal nerve were affected in most cases (82.35 and 66.01%, respectively), due to the compression from below, leaving the rostral V1 branch intact. VBD-related TN often showed multiple vessels contributing to the neurovascular conflict: in 30.39% of cases, an AICA+VBA combination was found, while SCA + VBA was found in 25.16% of patients.

### Clinical outcomes and surgical technique

Microvascular decompression (MVD) is the most effective surgical option for classic TN refractory to medical treatment (35–38). However, when the compression is secondary to VBA dolichoectasia, there is no universally accepted method for isolating the offending vessel. Generally, two approaches are performed: the interposition method and the transposition method. The transposition method theoretically reduces the risk of adhesion and granuloma formation at the decompression site, which are key factors in the recurrence of symptoms post-MVD. However, this method is often more time-consuming, complex, and potentially hazardous compared to the interposition method. The interposition method, which involves placing patches between the REZ and the offending vessels, is relatively straightforward and effective in relieving nerve compression. Despite some support for the interposition method, many experts believe it may result in inadequate decompression, thus diminishing the efficacy of MVD.

Our pooled analysis indicated that the transposition group had slightly higher rates of postoperative pain relief (considered as post-operative BNI score of 1–2) than the interposition group. However, this finding may be influenced by the higher heterogeneity of the interposition group in the included studies (I^2^ 59.5 and 27.5% in the interposition and transposition group, respectively, *p* = 0543). Indeed, the meta-regression analysis revealed an identical post-operative BNI group mean score (96%) for both techniques, suggesting they are comparable in terms of immediate pain relief.

In the study by Chai et al. (8), the authors compared the transposition and interposition techniques and found that the transposition group was associated with significantly better outcomes in terms of post-operative BNI score and pain-recurrence rate. They attributed these findings to several factors: given that in VBD the offending vessel is larger than AICA, SCA, and other vessels usually involved in classic TN, the interposition technique requires an increased amount of Teflon to achieve optimal decompression, posing a risk of applying additional pressure from excessive material used; additionally, the transposition technique can directly address the pulsatile transmission of the dolichoectatic VBA, which is believed to contribute to TN development and recurrence.

Despite these advantages, the transposition method is associated with higher postoperative complication rates. We found permanent facial hypoesthesia in 7% of cases and permanent hearing impairment in 8% at the last follow-up. It is also worth noting that the dolichoectatic arteries generally present with atherosclerosis, abnormal course, increased diameter, low elasticity, and limited mobility. These factors significantly increase the complexity of the surgery, particularly during vessel displacement, posing potential risks such as plaque dislodgement, rupture of branch vessels, and vasospasm (26).

### Strength and limitations

Our study has some limitations. The included articles were only small, retrospective, and single-institution case series and the reported data were incomplete in many of these. Follow-up ranged from 1 year up to 15 years, which may not provide a homogeneous long-term perspective. Additionally, the interposition group had fewer cases and greater heterogeneity across the included studies compared to the transposition group. This variability complicates direct comparison of data and necessitates caution when interpreting results. Despite these limitations, our study is the only meta-analysis comparing the interposition and transposition techniques for VBD-related TN, providing valuable insights into their relative efficacy and complications.

Future research should focus on larger, prospective studies with standardized reporting to better compare these techniques and refine surgical strategies to improve patient outcomes.

## Conclusion

Vascular compression is a frequent and treatable cause of essential trigeminal neuralgia. Even in the rare and complex case of a dolichoectatic vertebrobasilar artery, the microsurgical decompression method can provide excellent long-term outcomes in patients with TN who do not respond to medication. In our revision study, interposition and transposition groups revealed an identical post-operative BNI group mean score (96%) suggesting they are comparable in terms of immediate pain relief. However the transposition group is associated with higher postoperative complication rates.

## Data Availability

The original contributions presented in the study are included in the article/Supplementary material, further inquiries can be directed to the corresponding author.
