# Supplementary material for: Interposition versus transposition technique in microvascular decompression for trigeminal neuralgia secondary to vertebrobasilar dolichoectasia: a systematic review and pooled meta-analysis
Source: Front Neurol. 2024 Nov 25;15:1474553. doi: 10.3389/fneur.2024.1474553 (PMC11625671; doi:10.3389/fneur.2024.1474553)
Supplement: Supplementary file 1 [file Table_1.DOCX]

**Supplementary File 1**

**Search strategy**

Filters:

- Publication date: 1991 – 5/02/2024;
- Language: English

Database search:

- PubMed:
  - (((((((((((((((((((Trigeminal Neuralgia[MeSH Terms]) OR (Facial Neuralgia[MeSH Terms])) OR (Neuralgia, Trigeminal)) OR (Trigeminal Neuralgias)) OR (Tic Doloureux)) OR (Fothergill Disease)) OR (Disease, Fothergill)) OR (Tic Douloureux)) OR (Secondary Trigeminal Neuralgia)) OR (Neuralgia, Secondary Trigeminal)) OR (Secondary Trigeminal Neuralgias)) OR (Trigeminal Neuralgia, Secondary)) OR (Trigeminal Neuralgia, Idiopathic)) OR (Idiopathic Trigeminal Neuralgia)) OR (Idiopathic Trigeminal Neuralgias)) OR (Neuralgia, Idiopathic Trigeminal)))) AND ((((((((((Vertebrobasilar Insufficiency[MeSH Terms]) OR (Dolichoectasia, Vertebrobasilar)) OR (Vertebrobasilar Dolichoectasia)) OR (Dolichoectasias, Vertebrobasilar)) OR (Vertebrobasilar Dolichoectasias)) OR ("vertebrobasilar ectasia"[All Fields])) OR ("dolichoectatic basilar artery"[All Fields])) OR ("megadolichobasilar"[All Fields])) OR ("megadolichobasilarartery"[All Fields])) OR ("megadolichobasilar anomaly"[All Fields]))) AND ((((((Microvascular Decompression Surgery[MeSH Terms]) OR (Decompression Surgeries)) OR (Microvascular Decompression Surgery)) OR (Surgery, Microvascular Decompression)) OR (Microvascular Decompression)))
    - **38 results**
- Web of Science:
  - (((((((TS=(vertebrobasilar dolichoectasia)) OR TS=(vertebrobasilar ectasia)) OR TS=(dolichoectatic basilar artery)) OR TS=(megadolichobasilar)) OR TS=(megadolichobasilarartery)) OR TS=(megadolichobasilar anomaly)) AND TS=(microvascular decompression)) AND TS=(trigeminal neuralgia)
    - **60 results**
- Scopus:
  - vertebrobasilar AND dolichoectasia OR vertebrobasilar AND ectasia OR dolichoectatic AND basilar AND artery OR megadolichobasilar OR megadolichobasilarartery OR megadolichobasilar AND anomaly AND microvascular AND decompression AND trigeminal AND neuralgia
    - **56 results**
- Additional references:
  - **6 results**

**
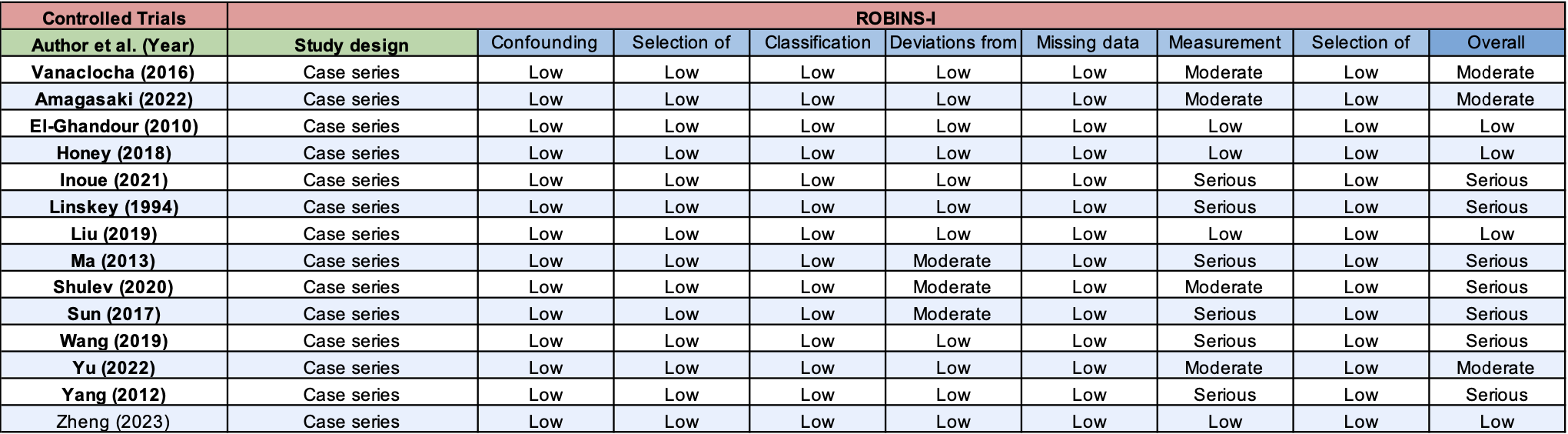
**

**Robins-I.**

**
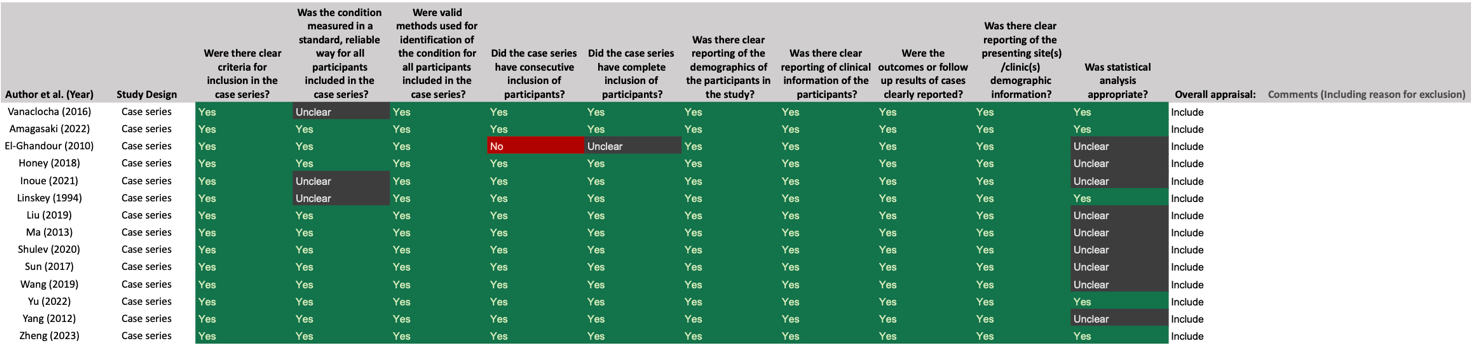
**

**JBI**
